# Supplementary material for: Bayesian design and analysis of two-arm cluster randomised trials using assurance: Extension to binary outcomes and comparison of Markov chain Monte Carlo and Integrated Nested Laplace Approximations
Source: Clin Trials. 2026 Mar 3;23(3):336–46. doi: 10.1177/17407745261421842 (PMC13242539; doi:10.1177/17407745261421842)
Supplement: sj-pdf-1-ctj-10.1177_17407745261421842 – Supplemental material for Bayesian design and analysis of two-arm cluster randomised trials using assurance: Extension to binary outcomes and comparison of Markov chain Monte Carlo and Integrated Nested Laplace Approximations [file sj-pdf-1-ctj-10.1177_17407745261421842.pdf]

Structured expert elicitation for design priors - the SPEEDY trial

Kevin Wilson, Abdullah Aloufi, Nina Wilson

1 Introduction

The SPEEDY trial is a two-arm cluster randomised controlled trial (RCT) with 1:1 randomisation between arms. The clusters are at the level of ambulance stations. There are two outcomes of interest, which were used to power the trial. One outcome is continuous (time to thrombectomy) and one is binary (proportion of patients undergoing thrombectomy). In this document we will outline the analysis in both the continuous and discrete cases for a cluster RCT, describe the process we will use to elicit design prior distributions for the SPEEDY trial and relate the elicited quantities to the design prior distributions of interest.

2 Analysis of cluster RCTs

2.1 Continuous outcome

If in the cluster RCT we observe continuous outcome Y_{ij} for individual $i = 1, \ldots, I_j$ in cluster $j = 1, \ldots, J$. We assume these are normally distributed $Y_{ij} \sim \mathrm{N}(\mu_j, \sigma_w^2)$, where σ_w^2 is the within cluster variance and the mean takes the form of a linear predictor

$$\mu_j = \lambda + \delta X_j + c_j.$$

The parameters in the linear predictor are the mean effect in the control arm λ, the treatment effect δ, a binary variable indicating whether cluster j is in the control arm $X_j = 0$ or treatment arm $X_j = 1$ and a random cluster effect $c_j \sim \mathrm{N}(0, \sigma_b^2)$ with between cluster variance σ_b^2. Standard prior distributions (there are other sensible choices) for the prior distributions in this case are

$$\begin{aligned} \lambda &\sim \mathrm{N}(m_\lambda, v_\lambda), \\ \delta &\sim \mathrm{N}(m_\delta, v_\delta), \\ \tau_w = \frac{1}{\sigma_w^2} &\sim \mathrm{Gamma}(a_w, b_w), \\ \tau_b = \frac{1}{\sigma_b^2} &\sim \mathrm{Gamma}(a_b, b_b). \end{aligned}$$

Additionally, we don't expect all of the clusters to be of equal size in SPEEDY. It is important to include this in the design of the trial, otherwise the trial could be underpowered. Suppose

the number of individuals recruited by the end of the trial to clusters $1, \dots, J$ are given by $\boldsymbol{n} = (n_1, \dots, n_J)$. We assume that this vector follows a multinomial distribution $\boldsymbol{n} \sim \text{Multinomial}(n_T, \boldsymbol{\theta})$, where $n_T = \sum_{j=1}^{J} n_j$ is the sample size in the trial and $\boldsymbol{\theta}$ is a vector containing the probabilities that a randomly selected individual from the trial would be in clusters $1, \dots, J$. A suitable prior distribution for $\boldsymbol{\theta}$ is

$$\boldsymbol{\theta} \sim \text{Dirichlet}(\boldsymbol{d}),$$

with hyperparameters $\boldsymbol{d} = (d_1, \dots, d_J)$.

The task for the prior elicitation is to ask an expert/group of experts questions which allow us to specify the hyperparameters $\boldsymbol{\psi}_{con} = (m_\lambda, v_\lambda, m_\delta, v_\delta, a_w, b_w, a_b, b_b, \boldsymbol{d})$. In the next section we will provide a list of elicitation questions for this model and in the following section we will describe how the answers to these elicitation questions define the hyperparameters in $\boldsymbol{\psi}_{con}$.

2.2 Discrete outcome

If in the cluster RCT we observe a binary outcome Y_{ij} for individual $i = 1, \dots, I_j$ in cluster $j = 1, \dots, J$, we can consider this a realisation from a Bernoulli random variable $Y_{ij} \sim \text{Bernoulli}(p_j)$, where p_j is the probability of a positive outcome in cluster j. One way to relate this to a linear predictor is via the logistic transformation, in which case,

$$\log\left(\frac{p_j}{1 - p_j}\right) = \lambda + \delta X_j + c_j.$$

As before the parameters in the linear predictor are the mean effect in the control arm λ, the treatment effect δ, a binary variable indicating whether cluster j is in the control arm $X_j = 0$ or treatment arm $X_j = 1$ and a random cluster effect $c_j \sim \text{N}(0, \sigma_b^2)$ with between cluster variance σ_b^2. We can use the same form for the prior distributions as in the continuous case

$$\begin{aligned} \lambda &\sim \text{N}(m_\lambda, v_\lambda), \\ \delta &\sim \text{N}(m_\delta, v_\delta), \\ \tau_b = \frac{1}{\sigma_b^2} &\sim \text{Gamma}(a_b, b_b). \end{aligned}$$

We have exactly the same form for the distribution of sample sizes in each cluster $\boldsymbol{n} \sim \text{Multinomial}(n_T, \boldsymbol{\theta})$ and the prior distribution on the vector of probabilities which must sum to one,

$$\boldsymbol{\theta} \sim \text{Dirichlet}(\boldsymbol{d}).$$

The set of hyperparameters for the elicitation in the discrete case is $\boldsymbol{\psi}_{dis} = (m_\lambda, v_\lambda, m_\delta, v_\delta, a_b, b_b, \boldsymbol{d})$. As in the continuous case, in the next section we will provide a list of elicitation questions for this model and in the following section we will describe how the answers to these elicitation questions define the hyperparameters in $\boldsymbol{\psi}_{dis}$.

3 Elicitation questions for Cluster RCTs

3.1 Continuous outcome

First we would like to elicit information which will allow us to specify the prior distribution for λ, the mean treatment effect in the control arm. This is an observable quantity in principle, and so we can ask about it directly. To understand the expert's uncertainty in this quantity we ask for the median and the upper and lower quartiles of the expert's distribution. That is, the elicitation questions in SPEEDY are:

1(a) *What is your median for the average time to thrombectomy in the control arm?*

1(b) *What is your lower quartile for the average time to thrombectomy in the control arm?*

1(c) *What is your upper quartile for the average time to thrombectomy in the control arm?*

In practice we use the SHELF protocol to structure the elicitation. This includes a step before 1. above, which elicits the expert's upper and lower plausible limits to reduce cognitive bias in the elicited judgements, and provides extensive guidance on how the expert should think about their median and quartiles, including steps challenging the values the expert chooses.

Next we require a prior distribution for the treatment effect, δ. We could ask about the difference in the mean time to thrombectomy between the two arms, but we choose the cognitively simpler task of asking the experts about the mean time to thrombectomy in the treatment arm. Specifically, we ask:

2(a) *What is your median for the average time to thrombectomy in the treatment arm?*

2(b) *What is your lower quartile for the average time to thrombectomy in the treatment arm?*

2(c) *What is your upper quartile for the average time to thrombectomy in the treatment arm?*

It is challenging for experts to think about standard deviations in time to thrombectomy between and within clusters, (σ_w, σ_b). Instead we will ask them about quantities that they have direct experience of observing in similar trials; the overall standard deviation in time to thrombectomy and the intra-class correlation (ICC), which measures the strength of the relationship in the time to thrombectomy between individuals in the same cluster. Specifically, we will ask the experts:

3(a) *What is your median for the standard deviation in time to thrombectomy?*

3(b) *What is your lower quartile for the standard deviation in time to thrombectomy?*

3(c) *What is your upper quartile for the standard deviation in time to thrombectomy?*

4(a) *What is your median for the ICC for time to thrombectomy?*

4(b) *What is your lower quartile for the ICC in time to thrombectomy?*

4(c) *What is your upper quartile for the ICC in time to thrombectomy?*

We have confirmed with the experts that the assumption that the standard deviations and ICCs will be equal in the two arms is reasonable.

This completes the elicitation questions for the continuous outcomes. In the next section we will demonstrate how to convert the answers to these questions into prior distributions on the model parameters.

3.2 Discrete outcome

We can ask similar questions to elicit (λ, δ) as in the continuous case. The main change is that we are now interested in proportions which are restricted to $[0, 1]$, rather than unbounded quantities. The questions 1(a)-1(c) and 2(a)-2(c) can be adjusted to:

6(a) *Consider an average cluster. What is your median for the proportion of patients who would receive a thrombectomy in the control arm?*

6(b) *Consider an average cluster. What is your lower quartile for the proportion of patients who would receive a thrombectomy in the control arm?*

6(c) *Consider an average cluster. What is your upper quartile for the proportion of patients who would receive a thrombectomy in the control arm?*

7(a) *Consider an average cluster. What is your median for the proportion of patients who would receive a thrombectomy in the treatment arm?*

7(b) *Consider an average cluster. What is your lower quartile for the proportion of patient who would receive a thrombectomy in the treatment arm?*

7(c) *Consider an average cluster. What is your upper quartile for the proportion of patients who would receive a thrombectomy in the treatment arm?*

The proportion $\bar{p}_1$ will be chosen to be a value consistent with the expert's answers to questions 6(a)-6(c) above. Similarly to questions 3(a)-3(c) and 4(a)-4(c) above, this is sufficient if we assume that the underlying correlation between clusters is consistent between arms.

4 The resulting priors

4.1 Continuous case

Denote the expert answers to questions 1(a)-1(c) above by $\boldsymbol{q}_C = (q_{0.25,C}, q_{0.5,C}, q_{0.75,C})$. These can be use to choose m_λ and v_λ, the mean and variance of the Normal prior for λ, via least squares fitting.

Denote the expert answers to questions 2(a)-2(c) above by $\boldsymbol{q}_T = (q_{0.25,T}, q_{0.5,T}, q_{0.75,T})$. These are quartiles of $\mu_T = \lambda - \delta$. If we use these values to fit a Normal distribution to μ_T (since the sum of Normal random variables is also Normal) with parameters (m_μ, v_μ) via

least squares, then we can find the mean and variance of δ as

$$\begin{aligned} m_\delta &= m_\lambda - m_\mu, \\ v_\delta &= v_\lambda - v_\mu. \end{aligned}$$

Denote the expert answers to questions 3(a)-3(c) and 4(a)-4(c) respectively as $(q_{0.25,\sigma}, q_{0.5,\sigma}, q_{0.75,\sigma})$ and $(q_{0.25,\rho}, q_{0.5,\rho}, q_{0.75,\rho})$. We can use these to fit hyperparameters of suitable priors $\sigma \sim \mathrm{Gamma}(a_\sigma, b_\sigma)$ and $\rho \sim \mathrm{Beta}(a_\rho, b_\rho)$ via least squares. Suppose we take samples from these distributions $(\sigma^{((k)}, \rho^{(k)})$ for $k = 1, \ldots, K$. The we can obtain samples

$$\begin{aligned} \sigma_b^{2(k)} &= \sigma^{2(k)} \rho^{(k)}, \\ \sigma_w^{2(k)} &= \sigma^{2(k)} (1 - \rho^{(k)}). \end{aligned}$$

We use the means and variances of these samples, $(\bar{m}_w, \bar{v}_w)$ and $(\bar{m}_b, \bar{v}_b)$ to specify the parameters of the prior gamma distributions of the between and within cluster variances as

$$a_b = \frac{m_b^2}{v_b}, \; b_b = \frac{m_b}{v_b},$$
$$a_w = \frac{m_w^2}{v_w}, \; b_w = \frac{m_w}{v_w}.$$

4.2 Discrete case

Denote the answers to questions 6(a)-6(c) by $(q_{0.25,C}, q_{0.5,C}, q_{0.75,C})$. These are quartiles for the distribution of p_j, where cluster j is in the control arm. We can use these to fit a prior Beta distribution for p_j via least squares. That is $p_j \sim \mathrm{Beta}(a_j, b_j)$, when cluster j is in the control arm. We can then take samples $p_j^{(k)}, k = 1, \ldots, K$ from this distribution, and then transform them via

$$\lambda_j^{(k)} = \log \left(\frac{p_j^{(k)}}{1 - p_j^{(k)}} \right).$$

Taking the mean and variance of the samples of $\lambda_j^{(k)}$ gives (m_λ, v_λ).

Denote the answers to questions 7(a)-7(c) by $(q_{0.25,T}, q_{0.5,T}, q_{0.75,T})$. As above, these are quartiles for the distribution of q_j, where cluster j is now in the treatment arm. We can use these to fit a prior Beta distribution for q_j via least squares. That is $q_j \sim \mathrm{Beta}(a_j, b_j)$, when cluster j is in the treatment arm. We can then take samples $q_j^{(k)}, k = 1, \ldots, K$ from this distribution, and then transform them via

$$\eta_j^{(k)} = \log \left(\frac{q_j^{(k)}}{1 - q_j^{(k)}} \right),$$

where now $\eta_j = \lambda + \delta$. Taking the mean and variance of the samples of $\eta_j^{(k)}$ gives (m_η, v_η) and, since λ and, the hyperparamters for δ can be found as

$$m_\delta = m_\eta - m_\lambda,$$
$$v_\delta = v_\eta - v_\lambda.$$

We then can elicit the varaince between σ_b^2 of the binary outcomes using the elicited distributions for $p_j \sim \mathrm{Beta}(a_j, b_j)$ the proportion in the control arm, $q_j \sim \mathrm{Beta}(a_j, b_j)$ the proportion in the treatment arm, and $\rho \sim \mathrm{Beta}(a_p, b_p)$ the ICC in the continuous case. The first step is to take the weighted average of the proportions samples $p_j^{(k)}$ and $q_j^{(k)}$, $k = 1, \ldots, K$ from that two distributions as

$$\pi^{(k)} = w_p p_j^{(k)} + w_q q_j^{(k)},$$

Since we have equal recruitment across arms then $w_p = w_q = 0.5$. Then, we use the weighted average samples $\pi^{(k)}$ and the samples from the ICC $\rho^{(k)}$ to elicit the variance between σ_b^2 as

$$\sigma_b^{2(k)} = \frac{\rho^{(k)}}{\pi^{(k)}[1 - \pi^{(k)}]},$$

We use the means and variances of this sample, $(\bar{m}_b, \bar{v}_b)$ to specify the parameters of the prior gamma distributions of the between cluster variance as

$$a_b = \frac{m_b^2}{v_b},\ b_b = \frac{m_b}{v_b}.$$
