# Supplementary material for: Bayesian design and analysis of two-arm cluster randomised trials using assurance: Extension to binary outcomes and comparison of Markov chain Monte Carlo and Integrated Nested Laplace Approximations
Source: Clin Trials. 2026 Mar 3;23(3):336–46. doi: 10.1177/17407745261421842 (PMC13242539; doi:10.1177/17407745261421842)
Supplement: sj-pdf-2-ctj-10.1177_17407745261421842 – Supplemental material for Bayesian design and analysis of two-arm cluster randomised trials using assurance: Extension to binary outcomes and comparison of Markov chain Monte Carlo and Integrated Nested Laplace Approximations [file sj-pdf-2-ctj-10.1177_17407745261421842.pdf]

Briefing document and evidence - SPEEDY expert elicitation exercise

Abdullah Aloufi, Kevin Wilson, Nina Wilson

Newcastle University

September 2024

This document provides information in advance for the elicitation session. Following a list of definitions, it defines the quantities of interest for the elicitation. It then summarises the main quantitative evidence available related to the quantities of interest. If you have further quantitative information which should be added to this document in advance of the elicitation session, please send it directly to Abdullah Aloufi, Nina Wilson or Kevin Wilson.

1 Definitions

SSNAP	Sentinel Stroke National Audit Programme [1].
Median	The middle value in a sorted list of outcomes where an outcome is equally likely to occur above or below.
Best Estimate	The median.
Lower 25% Quartile(Q_1)	Assuming the outcome will occur below the median, this is the value where an outcome is equally likely to occur above or below.
Upper 25% Quartile(Q_3)	Assuming the outcome will occur above the median, this is the value where an outcome is equally likely to occur above or below.
Intra-cluster correlation coefficient(ICC)	ICC is a measure of the relatedness of clustered data. It accounts for the relatedness of clustered data by comparing the variance within clusters with the variance between clusters.

2 Trial Summary

The SPEEDY trial [2] is a two-arm cluster randomised controlled trial (RCT) with 1:1 randomisation between standard care and the SPEEDY pathway. The clusters are at the level of the ambulance stations. The trial has co-primary outcomes of thrombectomy rate and time from stroke onset to thrombectomy (when thrombectomy is received). If the stroke symptom onset time is unclear the 'last known well' time will act as the onset time in keeping with standard clinical practice. Time of thrombectomy will be defined by the time of arterial puncture whether or not thrombus is subsequently removed.

The specialist prehospital redirection pathway (SPEEDY pathway) consists of:

1. Initiation by an ambulance practitioner using information routinely collected during suspected stroke assessment. Intervention ambulance practitioners will initiate the pathway if:

   - the incident is within a location where routine conveyance is to a Primary Stroke Centre which refers to a participating Comprehensive Stroke Centre (i.e. drip-ship

transfer area)

- the standard ambulance practitioner assessment indicates the following clinical criteria:
  - a clinical impression of suspected acute stroke with two symptoms of the standard FAST test both present: arm weakness and speech disturbance.
  - the symptoms began within the last 5 hours, or it is within 5 hours since waking with these symptoms.
  - a low risk of an endangered airway that would require additional urgent medical attention (Alert, Vocal response or Pain response (but not Unresponsive) on the AVPU scale).

2. Enhanced prenotification directly with the Comprehensive Stroke Centre instead of routine prenotification to the nearest local Primary Centre.

3. A remote specialist assessment undertaken by a member of the Comprehensive Stroke Centre team to make a decision about whether the patient should be redirected to the Comprehensive Centre or continue to the local Primary Centre.

The primary analysis population (PAP) is ambulance suspected stroke demonstrating pathway clinical criteria and usual conveyance would be to a Primary Stroke Centre. In addition, following hospital assessment a diagnosis of ischaemic stroke is assigned.

3 Quantities of Interest

The quantities of interest are the variables you will be asked about in the elicitation session. We require probability distributions on each of these quantities to carry out a Bayesian sample size calculation. The questions we will ask in the elicitation session will be repeated for each of the quantities of interest.

3.1 Time to thrombectomy

1. Average time to thrombectomy in the control arm.
2. Average time to thrombectomy in the intervention arm.
3. Standard deviation in time to thrombectomy.
4. Intra-cluster correlation coefficient (ICC) in time to thrombectomy.

3.2 Proportion of patients receiving thrombectomy

5 Proportion of patients who receive a thrombectomy in the control arm.

6 Proportion of patients who receive a thrombectomy in the intervention arm.

For all of the quantities of interest described above, we want to consider them within the primary analysis population.

4 Time from stroke onset to thrombectomy

In this section we present the main quantitative information available in relation to the quantities of interest 1-4 in Sections 4.1 - 4.4 respectively.

4.1 Time from stroke onset to thrombectomy in standard care

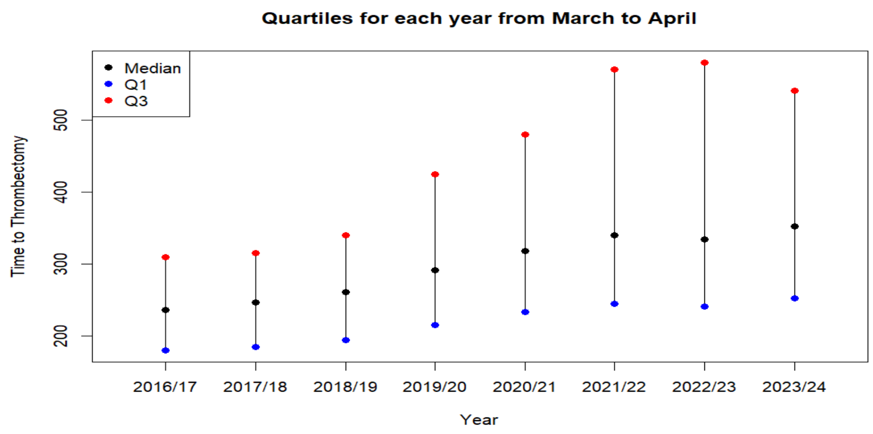

Figure 1: Time from onset to arterial puncture (min) using the SSNAP annual national thrombectomy reports from 2016-2024.

4.2 Time from stroke onset to thrombectomy in the treatment arm

This is defined as time from onset to arterial puncture (min) using the SPEEDY specialist prehospital redirection pathway. We have used the stroke onset to arterial puncture times from SSNAP as plotted in Figure 1 and assumed a time reduction (treatment effect/ minimum clinically important difference) of 30 minutes to calculate a hypothesised time from onset to arterial puncture (min) using the SPEEDY pathway.

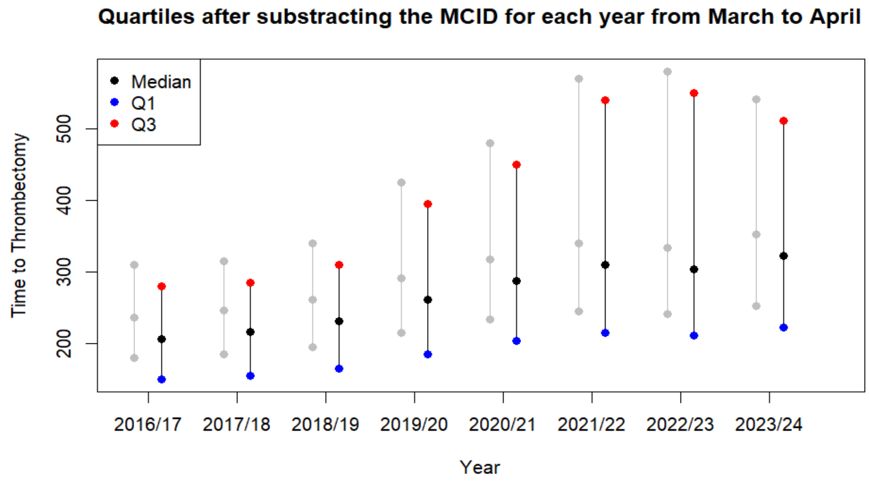

Figure 2: Time from onset to arterial puncture (min) using the SPEEDY specialist prehospital redirection pathway assuming a 30 minute reduction from the SSNAP data presented in Figure 1.

4.3 The standard deviation of the time from stroke onset to thrombectomy

In the SPEEDY trial, the sample size calculation used a standard deviation of 120 for the time from onset to thrombectomy. We have calculated the standard deviation of the onset from the SSNAP data for the years 2016-2024 in Figure 3, using an approximation formula $\sigma \approx \frac{IQR}{1.349}$, where, $IQR = Q_3 - Q_1$ is the interquartile range. For this approximation we need to meet a normality assumption.

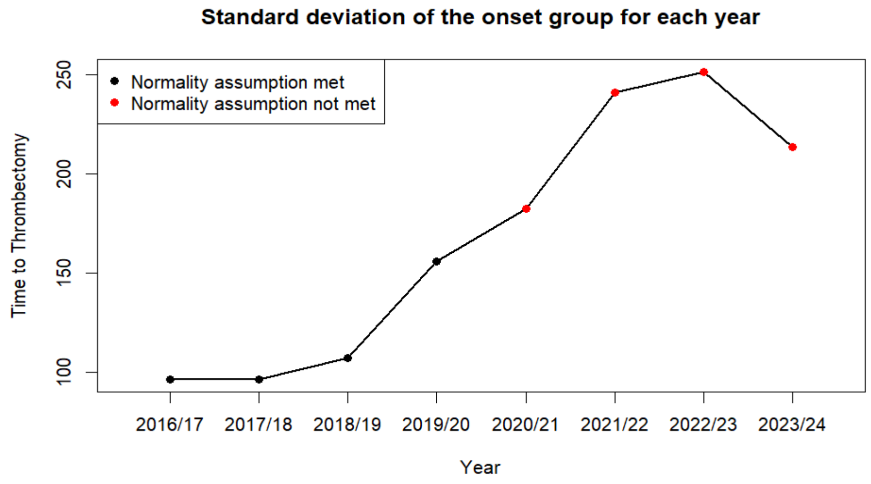

Figure 3: The standard deviation of the onset to arterial puncture time (min) calculated from the SSNAP national annual thrombectomy reports from 2016 to 2024.

4.4 The intra-cluster correlation coefficient (ICC) for time from stroke onset to thrombectomy

The sample size calculation for SPEEDY assumed an ICC of 0.01. Below we have summarised ICCs from trials in similar settings.

4.4.1 SAFER Trial

The Support and Assessment for Fall Emergency Referrals (SAFER) 2 trial [4] is a cluster randomised trial of the clinical effectiveness of new protocols for emergency ambulance paramedics to assess older people following a fall with referral to community-based care when appropriate. The clusters were ambulance stations. The primary outcome comprised subsequent emergency health-care contacts (death, emergency admissions, emergency department (ED) attendances and emergency service calls) at 1 month and 6 months. Between March 2011 and June 2012 a total of 5914 eligible patients were attended by 215 paramedics based at 25 ambulance stations. The trial profile is given in Figure 6. The ICCs for the primary outcome and its components at 6 months are given in Table 1.

Table 1: Primary outcome components at 6 months analysed by intervention group.

Primary outcome	Raw data: Intervention	Raw data: Control	Adjusted comparison[a,b]: Estimate, p-value	Adjusted comparison[a,b]: 95% CI	ICC: Estimate	ICC: 95% CI
Overall composite outcome						
Proportion of patients with further emergency service call, ED attendance, emergency admission or death,[c] *n/N* (%)	1701/2391 (71.1)	1592/2264 (70.3)	OR = 1.018; $p = 0.789$	0.895 to 1.157	0	n/a
Primary outcome components						
Proportion of patients dying (any cause),[d] *n/N* (%)	458/2391 (19.2)	419/2264 (18.5)	OR = 1.187; $p = 0.094$	0.971 to 1.451	0	n/a
Proportion with further emergency admission,[e] *n/N* (%)	1153/2391 (48.2)	1084/2264 (47.9)	OR = 1.001; $p = 0.984$	0.891 to 1.125	0.0052	0.0016 to 0.0113
Proportion of patients with further ED attendance,[f] *n/N* (%)	1079/2391 (45.1)	1021/2264 (45.1)	OR = 0.999; $p = 0.986$	0.888 to 1.123	0.0191	0.0085 to 0.0342
Further ED attendances per patient,[g] mean (SD) [*n*]	0.844 (1.392) [2380]	0.913 (2.738) [2257]	$\Lambda = 0.810$; $p < 0.001$	0.722 to 0.909	0.0144	0.0062 to 0.0261
Further ED attendances/patient/day at risk,[h] mean (SD) [*n*]	0.0169 (0.0907) [2380]	0.0144 (0.0686) [2257]	$\Delta = 0.0025$; $p = 0.292$	−0.0021 to 0.0071	0.0041	0.0011 to 0.0098
			$\Delta_L = -0.0163$; $p = 0.711$	−0.1024 to 0.0699	0.0264	0.0127 to 0.0451
Proportion of patients with further emergency service call,[i] *n/N* (%)	1046/2391 (43.7)	1046/2264 (46.2)	OR = 0.899; $p = 0.076$	0.799 to 1.011	0.0030	0.0006 to 0.0079
Further emergency service calls per patient,[j] mean (SD) [*n*]	1.136 (2.506) [2380]	1.251 (2.672) [2257]	$\Lambda = 0.931$; $p = 0.076$	0.860 to 1.007	0.0040	0.0012 to 0.0090
Further emergency service calls/patient/day at risk,[k] mean (SD) [*n*]	0.0125 (0.0363) [2380]	0.0172 (0.0599) [2257]	$\Delta = -0.0045$; $p = 0.002$	−0.0073 to -0.0017	0.0029	0.0004 to 0.0069
			$\Delta_L = -0.1183$; $p = 0.010$	−0.2079 to −0.0286	0.0036	0.0009 to 0.0086

4.4.2 PASTA Trial

The PASTA trial [3] is a cluster randomised controlled clinical trial to determine whether a Paramedic Acute Stroke Treatment Assessment (PASTA) pathway can speed up access to emergency stroke treatments, especially thrombolysis treatment, and so improve recovery after stroke. Clusters were individual paramedics based within pre-randomised ambulance stations. Participants receive either usual emergency stroke care or the additional PASTA pathway. The primary outcome was the proportion of patients receiving thrombolysis. Patients were recruited between December 2015 and July 2018. The sample size calculation based on the primary outcome of thrombolysis rate used an ICC of 0.02. The analysis of this outcome at the end of the trial reported an ICC of 0.00.

5 Thrombectomy rate

In this section we present the main quantitative information available for quantities of interest 5-6 in Sections 5.1-5.2.

5.1 Proportion of patients who have a thrombectomy

The SPEEDY trial assumed a conservative baseline thrombectomy rate of 3% in the total stroke population across centres, a minimal important absolute change in procedures would be 2%. This is equivalent to 13.2% with a change of 8.4% amongst the primary analysis population. We use the proportion of patients who have a thrombectomy in the total stroke population across centres from [1] in Figure 4, to calculate the equivalent proportion of patients who have a thrombectomy in the primary analysis population in Figure 5.

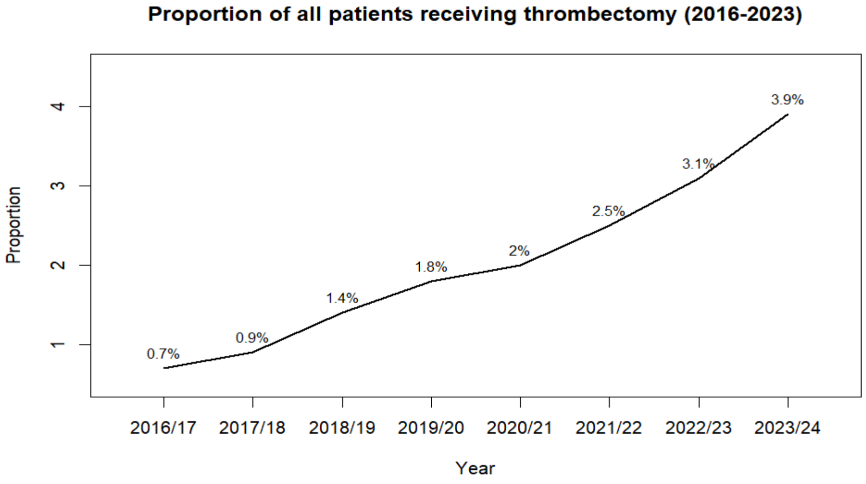

Figure 4: Proportion patients who have thrombectomy in the total stroke population across centres using the SSNAP annual national thrombectomy reports from 2016-2024.

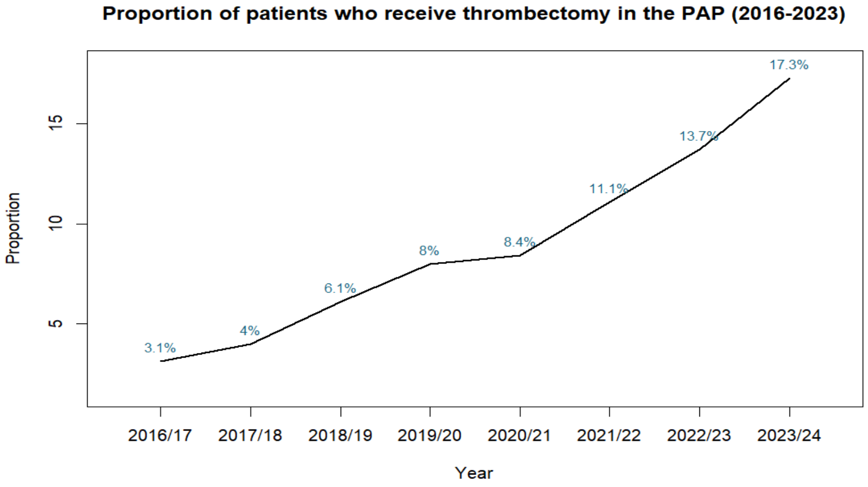

Figure 5: Proportion of patients who receive thrombectomy in the primary analysis population (PAP) calculated from SSNAP in Figure 4 for the years 2016-2024.

References

[1] Sentinel Stroke National Audit Programme. School of Population Health and Environmental Studies King's College London. https://www.strokeaudit.org.

[2] SPEEDY trial protocol version 2 IRAS ID 312053, (2022).

[3] Price CI, White P, Balami J, Bhattarai N, Coughlan D, Exley C, et al. Improving emergency treatment for patients with acute stroke: the PEARS research programme, including the PASTA cluster RCT. Programme Grants Appl Res 2022;10(4)

[4] Snooks HA, Anthony R, Chatters R, Dale J, Fothergill R, Gaze S, et al. Support and Assessment for Fall Emergency Referrals (SAFER) 2: a cluster randomised trial and systematic review of clinical effectiveness and cost-effectiveness of new protocols for emergency ambulance paramedics to assess older people following a fall with referral to community-based care when appropriate. Health Technol Assess 2017;21(13).
