# Supplementary figures and images for: Bayesian design and analysis of two-arm cluster randomised trials using assurance: Extension to binary outcomes and comparison of Markov chain Monte Carlo and Integrated Nested Laplace Approximations
Source: Clin Trials. 2026 Mar 3;23(3):336–46. doi: 10.1177/17407745261421842 (PMC13242539; doi:10.1177/17407745261421842)

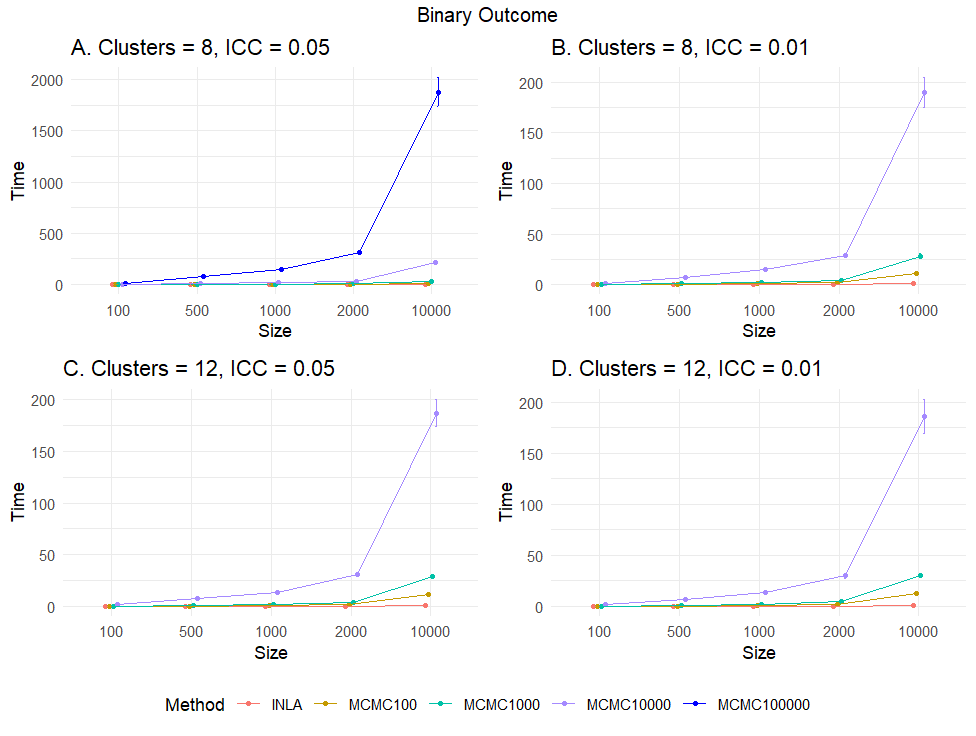

Supplement: sj-png-3-ctj-10.1177_17407745261421842 – Supplemental material for Bayesian design and analysis of two-arm cluster randomised trials using assurance: Extension to binary outcomes and comparison of Markov chain Monte Carlo and Integrated Nested Laplace Approximations [file sj-png-3-ctj-10.1177_17407745261421842.png]

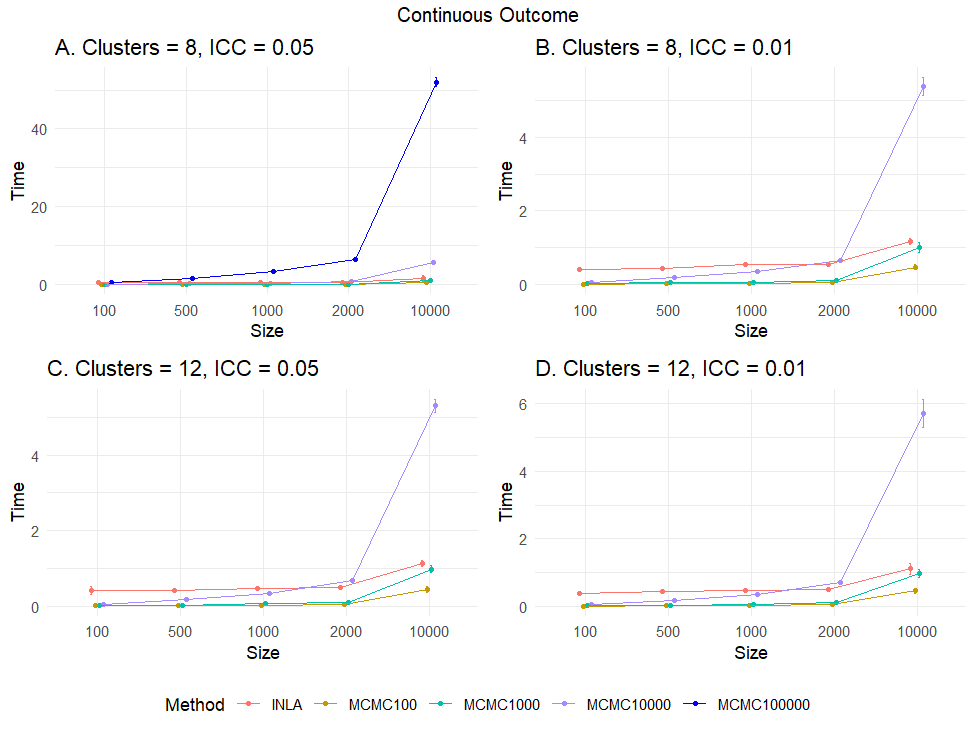

Supplement: sj-png-4-ctj-10.1177_17407745261421842 – Supplemental material for Bayesian design and analysis of two-arm cluster randomised trials using assurance: Extension to binary outcomes and comparison of Markov chain Monte Carlo and Integrated Nested Laplace Approximations [file sj-png-4-ctj-10.1177_17407745261421842.png]

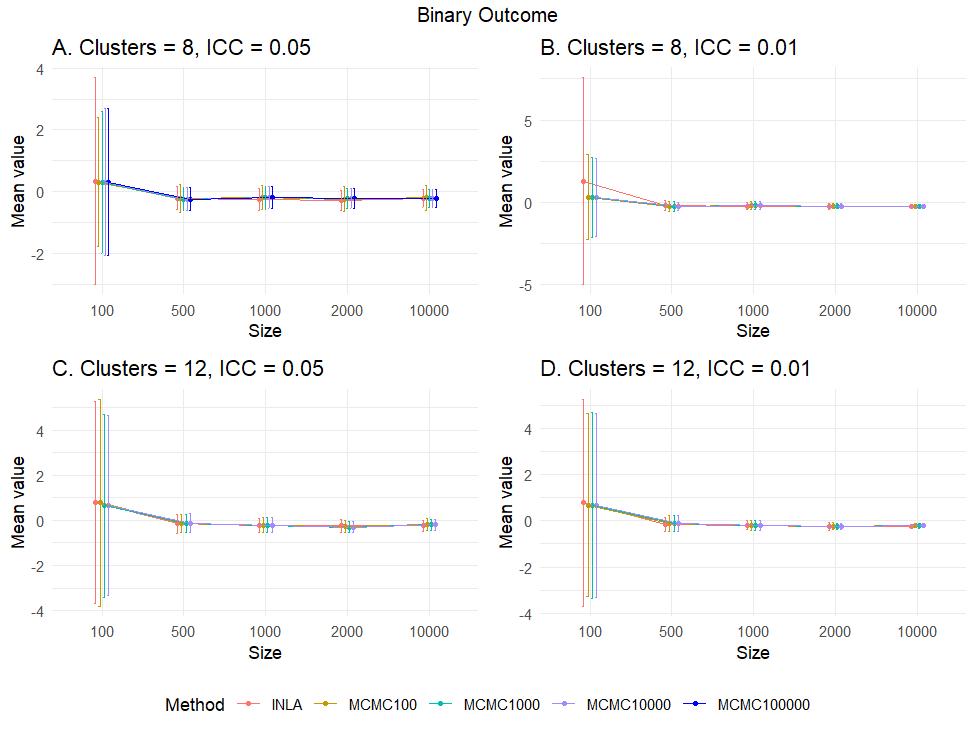

Supplement: sj-png-5-ctj-10.1177_17407745261421842 – Supplemental material for Bayesian design and analysis of two-arm cluster randomised trials using assurance: Extension to binary outcomes and comparison of Markov chain Monte Carlo and Integrated Nested Laplace Approximations [file sj-png-5-ctj-10.1177_17407745261421842.png]

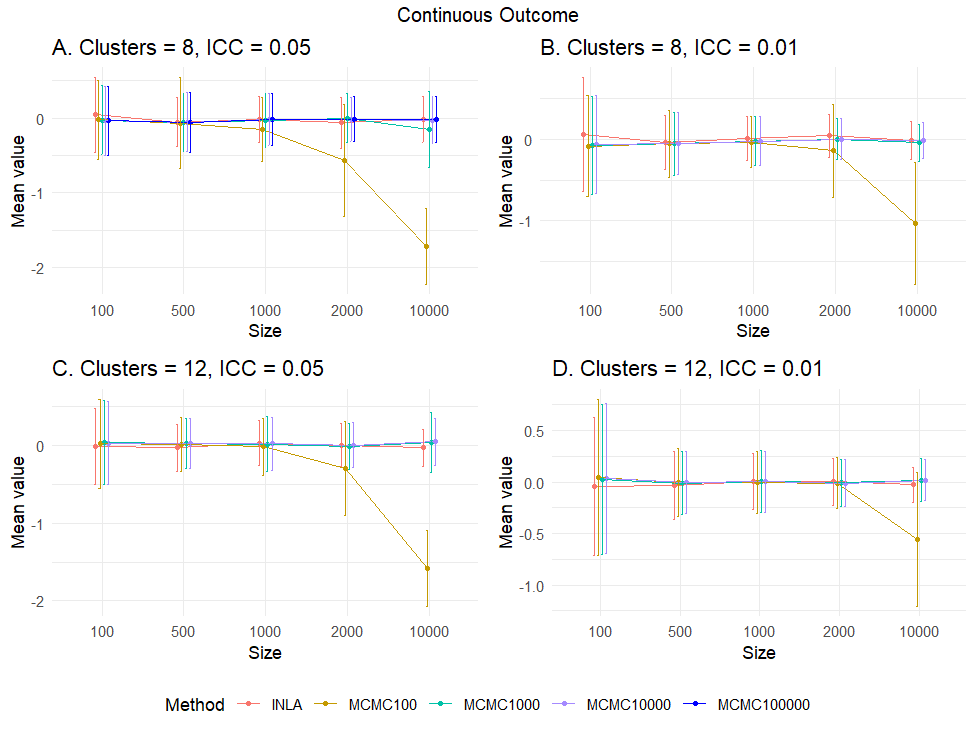

Supplement: sj-png-6-ctj-10.1177_17407745261421842 – Supplemental material for Bayesian design and analysis of two-arm cluster randomised trials using assurance: Extension to binary outcomes and comparison of Markov chain Monte Carlo and Integrated Nested Laplace Approximations [file sj-png-6-ctj-10.1177_17407745261421842.png]
